# Retrogradation of Maize Starch after High Hydrostatic Pressure Gelation: Effect of Amylose Content and Depressurization Rate

**DOI:** 10.1371/journal.pone.0156061

**Published:** 2016-05-24

**Authors:** Zhi Yang, Peter Swedlund, Qinfen Gu, Yacine Hemar, Sahraoui Chaieb

**Affiliations:** 1 School of Chemical Sciences, University of Auckland, Private Bag 92019, Auckland 1142, New Zealand; 2 Australian Synchrotron, 800 Blackburn Rd., Clayton 3168, Australia; 3 Division of Biological and Environmental Science and Engineering, KAUST, Thuwal, 23955, KSA; 4 Lawrence Berkeley National Laboratory, 1 Cyclotron Road, Mailstop 6R-2100. Berkeley, CA, 94720, United States of America; Institute for Frontier Medical Sciences, Kyoto University, JAPAN

## Abstract

High hydrostatic pressure (HHP) has been employed to gelatinize or physically modify starch dispersions. In this study, waxy maize starch, normal maize starch, and two high amylose content starch were processed by a HHP of the order of 600 MPa, at 25°C for 15min. The effect of HHP processing on the crystallization of maize starches with various amylose content during storage at 4°C was investigated. Crystallization kinetics of HHP treated starch gels were investigated using rheology and FTIR. The effect of crystallization on the mechanical properties of starch gel network were evaluated in terms of dynamic complex modulus (G*). The crystallization induced increase of short-range helices structures were investigated using FTIR. The pressure releasing rate does not affect the starch retrogradation behaviour. The rate and extent of retrogradation depends on the amylose content of amylose starch. The least retrogradation was observed in HHP treated waxy maize starch. The rate of retrogradation is higher for HHP treated high amylose maize starch than that of normal maize starch. A linear relationship between the extent of retrogradation (phase distribution) measured by FTIR and G* is proposed.

## Introduction

Starch granules are semi-crystalline and are composed mainly of a mixture of two α-D-glucose polymers; amylose and amylopectin. The crystalline regions in the granules are mainly due to the presence of clusters of amylopectin, which is a highly branched polymer of several million Daltons. Amylose is predominantly made up of linear starch chains and is largely amorphous and randomly distributed between the amylopectin clusters [1]. When the starch granule is heated in the presence of water, the semi-crystalline structures is lost and transformed, by a heat-induced starch gelatinization, into an amorphous form [2–5]. During storage, gelatinized starch tends to re-assemble, through starch retrogradation, into ordered crystalline structures[6].

In recent years high hydrostatic pressure (HHP) has been used extensively to physically modify and gelatinize starches from different botanical sources [7–9]. The starch gelatinization characteristics, morphology changes, rheological properties and other physicochemical properties have been extensively studied [10–13]. However, few studies have been conducted on the retrogradation properties of pressure-gelatinized starch [14]. Starch retrogradation is a multistage process in which the organization of amylose dominates the short-term retrogradation, whereas the long-term development of crystallinity is dominated by changes in the amylopectin. The effect of retrogradation on starch may be desirable (for production of resistant starch) or undesirable [15]. Starch retrogradation has a complex dependence on many variables. These include starch granule size [16], starch concentration [17], storage time and temperature [18, 19], amylose content [20–22], phosphorus content [21], co-crystallization of amylose with amylopectin [23], the presence of nonstarch components such as lipids [24], proteins [25], and oligosaccharides [26], [2, 27]. Understanding the kinetics of starch retrogradation and the properties of high pressure treated starch is both scientifically interesting and extremely important for many commercial applications.

Previous studies on the retrogradation of HHP treated starches have used waxy and normal starch with low amylose content [14, 28]. The pressure applied can cause full gelatinization of the starch and the retrogradation can then be studied following depressurization. However, high amylose maize starch (B-type) is more resistant to HHP and only partial gelatinization occurs during HHP treatment. However, there are no reports regarding the effect of amylose/amylopectin ratio on the retrogradation kinetics of maize starch HPP gels. Another important variable influencing post HHP treatments is the rate of pressure release. For example it is suggested that the pressure release rate influences both casein micelles aggregation [29] and whey proteins denaturation [30] in dairy systems treated by HHP. Therefore, the objectives of this work were: to investigate the retrogradation properties of maize starch with various amylose/amylopectin ratios after HHP treatment, to explore the effect of amylose content on the retrogradation of these gels and to determine the effect of the rate of pressure release on the retrogradation of HHP gelled starch dispersions.

## Materials and Methods

### Materials

Waxy (MAZACA), Normal maize starch (Melojel), and high-amylose starch (Gelose50 and Gelose80) maize starch powders were donated by National Starch Food Innovation (Auckland, New Zealand). Their chemical composition are reported in Table 1.

**Table 1:** Chemical composition (w/w, %) of the maize starches used.

	Waxy starch (MAZACA)	Normal starch (Melojel)	Gelose50	Gelose80
Amylose	3.59±0.29	29.6±0.7	52.7±0.4	89.78±0.960
Moisture	10.4±0.2	8.32±0.01	10.31±0.06	12.29±0.690
Lipid	0.15±0.02	0.11±0.02	0.35±0.09	0.140±0.003
Protein	0.14±0.01	0.16±0.03	0.46±0.11	2.66±0.40
Ash	0.06±0.01	0.11±0.01	0.12±0.04	0.100±0.010

### Preparation of starch suspensions

Starch was suspended in 18.2 MΩ cm Milli-Q water at a concentration of 10 w/w%. Sodium Azide (Sigma Aldrich, USA) was added at a concentration of 0.02 w/w% to the starch suspension to eliminate mold growth during storage. Beckman Polyallomer centrifuge tubes (25 mm internal diameter × 64 mm height, Beckman Instruments, Inc., USA) were filled with ≈10 mL starch suspension and heat sealed prior to high pressure treatment.

### High pressure treatment

Pressure treatments were conducted using a laboratory-scale high-pressure unit (Stansted mini Food Lab, Stansted Fluid Power Ltd., Stansted, UK), at pressures of 600 MPa, for 15 min at 25°C. The pressurization rate was set at 900 MPa/min and the depressurization rates investigated were 50 MPa/min, 100 MPa/min, and 900 MPa/min. Once the high-pressure treatment was completed, the sample tubes were opened and the samples transferred into plastic 15 mL centrifuge tubes. If a sediment was present, it was mixed thoroughly by a vortex mixer (KIA, Germany) to ensure sample homogeneity. The samples were then kept in closed tubes at 4°C and analysed as a function of storage time.

### Rheological properties

The rheological properties of the pressure treated and control corn starch (non-high pressure treated) were studied as a function of time at storage temperature of 4°C using a stress-controlled rheometer (MCR 302, Anton Paar Austria). Dynamic rheological measurements study viscoelastic behaviour of HHP treated corn starch pastes and controlled starch suspensions. A 25 mm diameter plate and plate geometry, with a gap of 1.0 mm, was employed to perform dynamic rheological tests.

The different stages of heating, cooling and rheological measurements were as follow: In s*tep 1* the sample was transferred to the rheometer plate preheated to 50°C and held for 5 min without shear to achieve temperature equilibrium. In *step 2* the sample was heated from 50 to 95°C, at a heating rate of 3°C/min at a strain of 1% and 1 Hz frequency. In s*tep 3* the sample was held at 95°C for 5 min with strain of 1% and 1 Hz frequency. In s*tep 4* the sample was cooled from 95 to 25°C, at a rate of 3°C/min at a strain of 1% and 1 Hz frequency. After the temperature reached 25°C and was held there for 5 min, a dynamic frequency sweep (small-deformations) measurement was performed, followed by *step 5* where a dynamic strain-sweep (large deformations) was performed to ensure that the frequency sweep was within the linear viscoelastic region. The frequency sweep measurement (*step 4*) was carried out at a constant strain of 1% for frequencies ranging from 0.01 Hz to 1 Hz, and the strain-sweep measurements (*step 5*) was performed at a constant frequency of 1 Hz from 0.1% to 10,000%. In these dynamic measurements the elastic modulus *G’*, the viscous modulus *G”* and the complex modulus G* given by |G*| = (G’^2^+G”^2^)^1/2^, were obtained.

### Fourier transform infra-red spectroscopy (FTIR)

FTIR spectra were obtained on a PerkinElmer Spectrum 100 Spectrometer (U.K.) fitted with DTGS (deuterated triglycine sulfate) detector and a Universal Attenuated Total Reflectance (ATR) single reflectance cell with a diamond crystal. Following HHP and after various storage time at 4°C, starch samples were freeze-dried and ground to a powder for FTIR analysis. Starch samples were placed on the surface of the crystal. For each spectrum 64 scans were co-added at a resolution of 4 cm^-1^ using the empty cell as background. Duplicate measurements were conducted for each sample. Data analysis was carried out using the OMNIC 6.0 software (Thermo Electron Corporation, USA). Spectra were linear baseline-corrected between 1200 and 800 cm^-1^ and deconvoluted to resolve overlapped bands [31]. A half-bandwidth of 26 cm^-1^ and an enhancement factor of 2.4 with triangular apodization was employed according to [32]. Intensity measurements were performed on the deconvoluted spectra by recording the height of the absorbance bands from the baseline. The ratios of absorbance height 1045 cm^-1^/1022 cm^-1^ were obtained from the deconvoluted spectra.

## Results and Discussion

**Fig 1:**
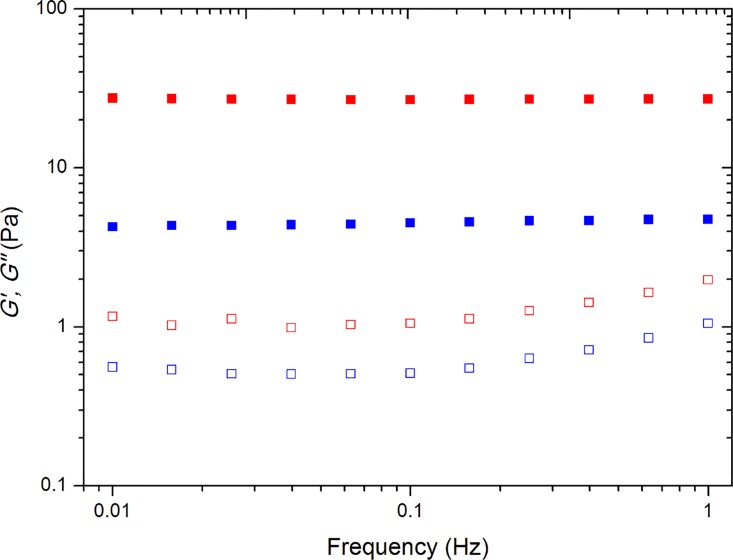
Storage modulus *G’* (solid symbols) and loss modulus *G”* (open symbols) as a function of frequency. Symbols are Gelose 80 starch after HHP treatment (pressure releasing rate 100MPa/min) for 0 day storage (blue) and 30 days storage (red).

**Fig 2:**
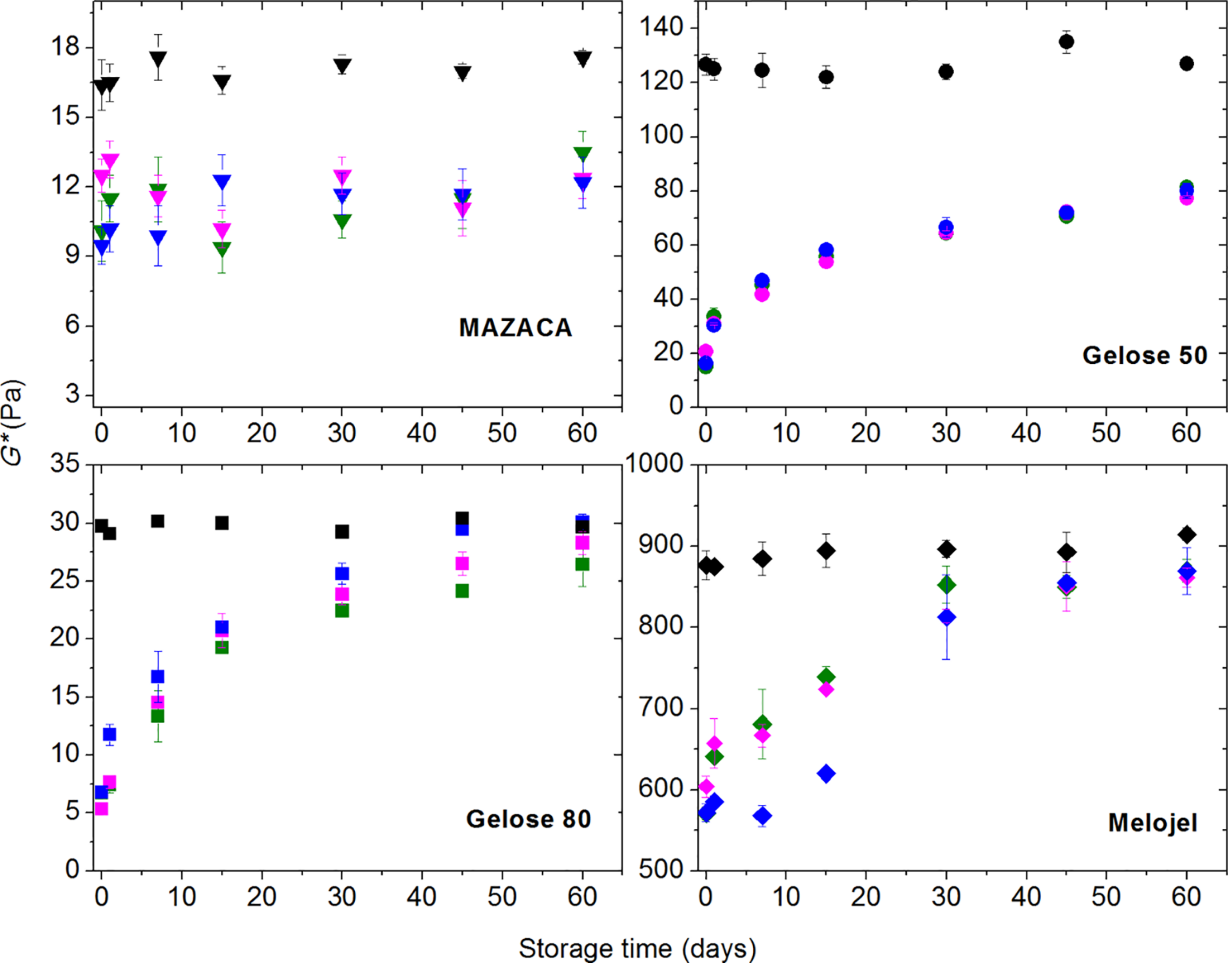
Complex modulus (G*) at 1 Hz, for Gelose 50, Gelose 80, MAZACA, and Melojel as a function of time for storage starch pastes stored at 4°C. Black: Non-treated control sample, Olive: Pressure releasing rate: 50 MPa/min, Magenta: Pressure releasing rate: 100 MPa/min, Blue: Pressure releasing rate: 900 MPa/min.

**Fig 3:**
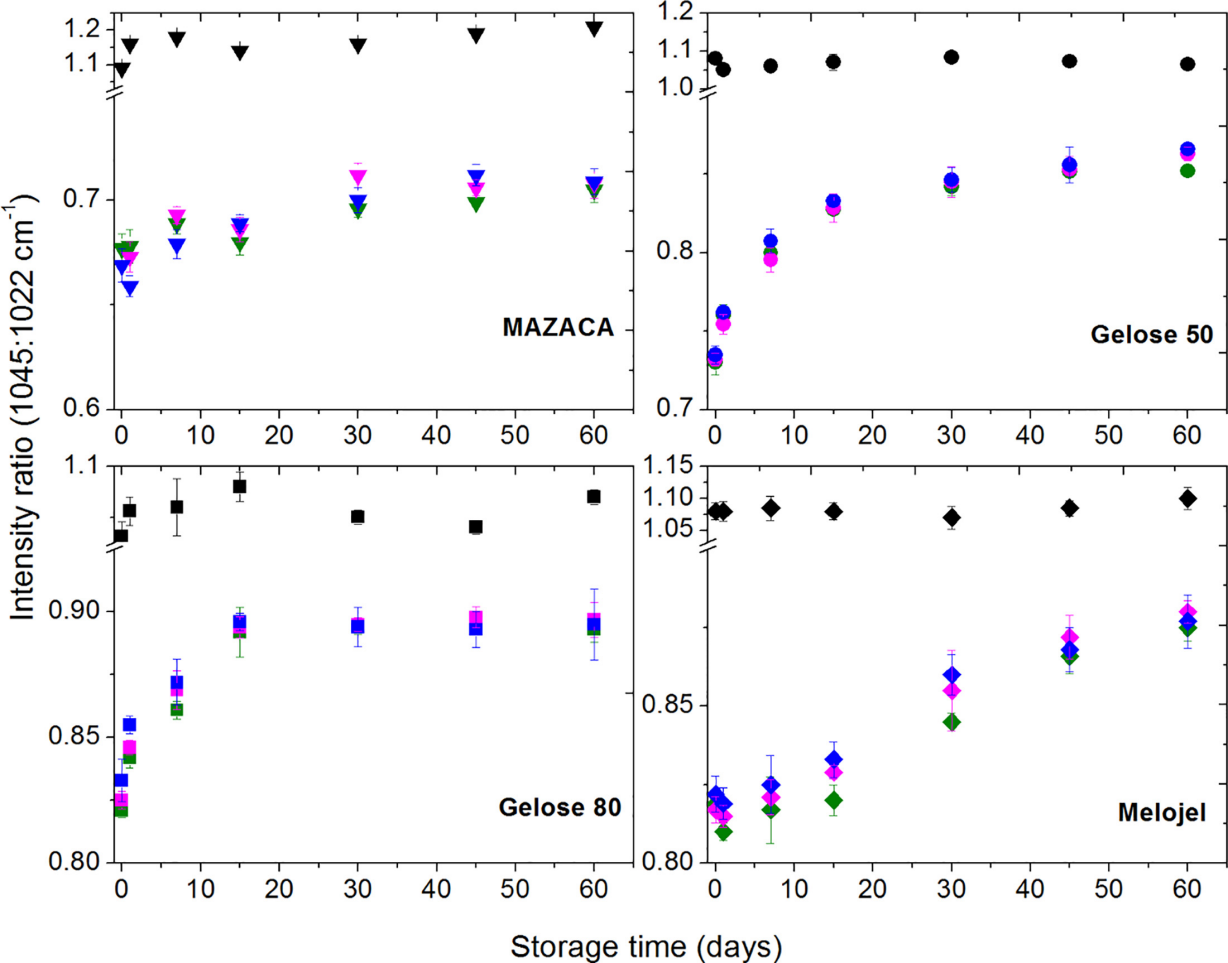
Retrogradation monitored using the FTIR absorbance ratio 1045:1022 cm^-1^ for Gelose 50, Gelose 80, MAZACA, and Melojel. Black: Non-treated control sample, Olive: Pressure releasing rate: 50 MPa/min, Magenta: Pressure releasing rate: 100 MPa/min, Blue: Pressure releasing rate: 900 MPa/min.

A strain sweep at frequency of 1 Hz was conducted to determine the linear viscoelastic region for all HHP processed starch samples as a function of storage time. A frequency sweep (Fig 1) was performed at a constant strain of 1%, which falls into the linear viscoelastic region. The complex modulus G* was calculated at 1 Hz for all HHP treated maize starch and are shown in Fig 2 as a function of storage time. For non-HHP treated control samples, normal maize starch (Melojel) demonstrates the highest G* value after pasting, while waxy (MAZACA) and high amylose (Gelose 50 and Gelose 80) showed much lower G* value. It is well known that such high amylose starch have an elevated temperature of crystalline dissociation, preventing the onset swelling below 90°C [33]. The lower G* value of waxy maize starch could be due to the lack of dissolving amylose molecules, which may be attributed to the formation of a network of swollen starch granules [34]. For all HHP-treated maize starch, the G* value is lower than that of non-HHP-treated starch. This could be due to the excessive pressurization weakening starch gel structures [35, 36]. For all HHP-treated maize starch except MAZACA, the G* values first increase and then level out into a plateau. For retrogradation of amylose in high amylose content maize starch, it has been suggested that rapid formation of a cross-linked network arises from the adoption of ordered double-helical chain segments, acting as “junction zones,” which are interconnected by more mobile amorphous single-chain segments [37]. On the other hand, amylopectin gelation in waxy maize starch is a slow process involving intra- and inter-molecular chain associations [38]. For all maize starch, the rate of pressure release after HPP did not greatly influence the retrogradation behaviour as measured by G*. HHP release rate was found to have an effect on milk protein denaturation and subsequent aggregation (Merel-Rausch et al. 2007; Devi et al., 2013). In the case of starch, denaturation is not expected, and the rate of retrogradation is much slower as can be seen in Figs 2 and 3, compared to the rate of pressure release which is up to 12 min (for the lowest pressure releasing rate: 50 MPa/min).

**Fig 4:**
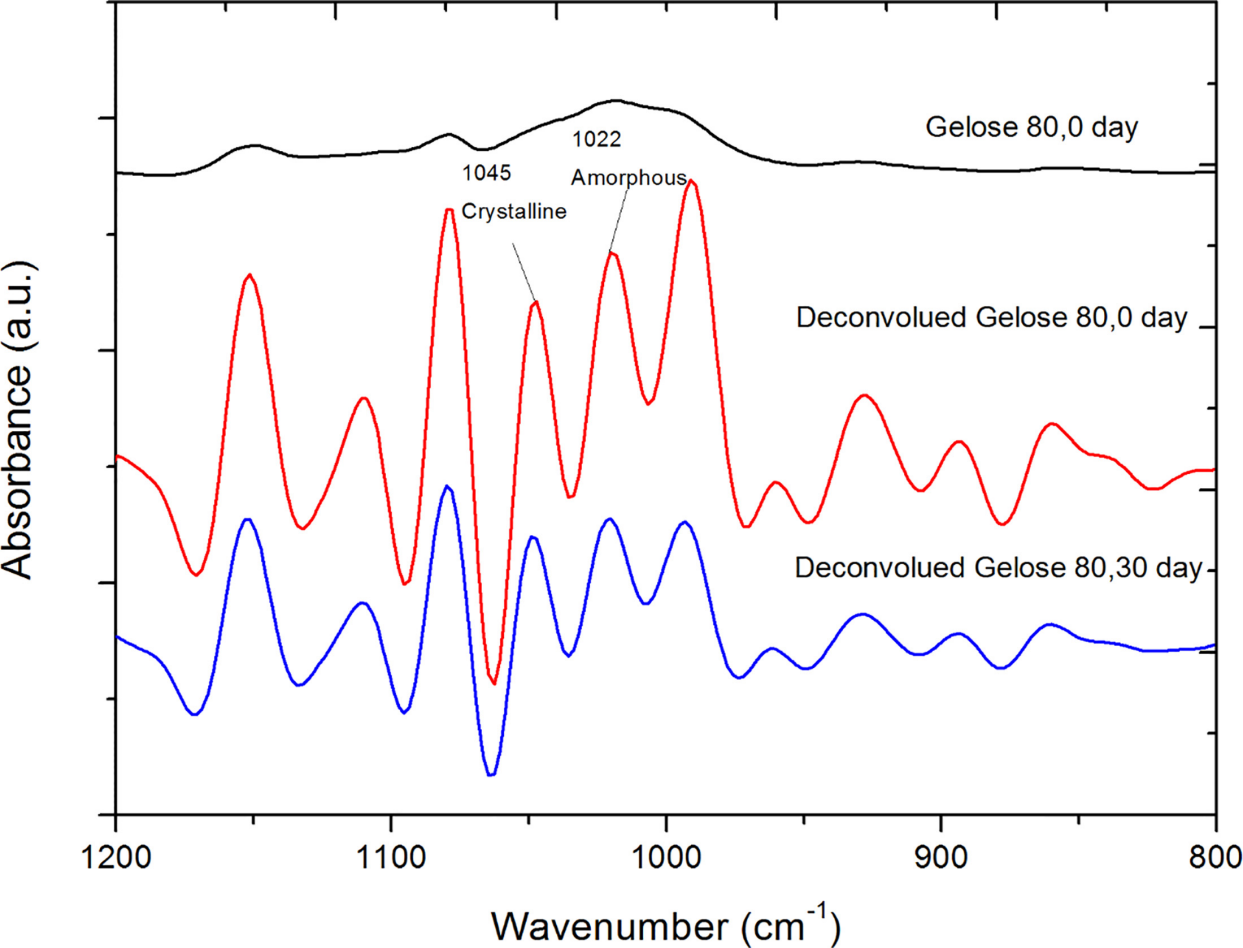
The original and deconvoluted FTIR spectrum of maize starch Gelose 80 after 0 and 30 days storage after HHP treatment (pressure releasing rate: 100MPa/min).

FTIR spectra were measured to determine the extent of short-range molecular order (helical order) in these high pressure-treated starch samples during storage (Figs 3 and 4). We wish to provide molecular insights into the connections between the degree of order and the network mechanical behaviour as determined by rheology. The bands at 1045 cm^-1^ and 1022 cm^-1^ have been assigned to the ordered and amorphous phases of starch, respectively [27, 39, 40]. The ratios of the heights of the bands at 1045 and 1022 cm^-1^ will therefore be related to the ratio of ordered starch to amorphous starch [41]. For all HHP-treated maize starch, the ratio of bands at 1045 and 1022 cm^-1^ is lower than that of their non-HHP treated counterparts. This is due to a decrease of the crystalline phase size concomitant with an increase of the amorphous phase size caused by HHP induced starch gelatinization [42].

The FTIR spectra are strongly correlated with the rheology in terms of the retrogradation kinetics and the degree of retrogradation (Fig 3). Both measurements show that Gelose 50 and Gelose 80 have the fastest rate of retrogradation, followed by Melojel and MAZACA demonstrating the lowest rate of retrogradation. The variations in the above changes among the high pressure treatments with different pressure releasing rate were marginal.

**Fig 5:**
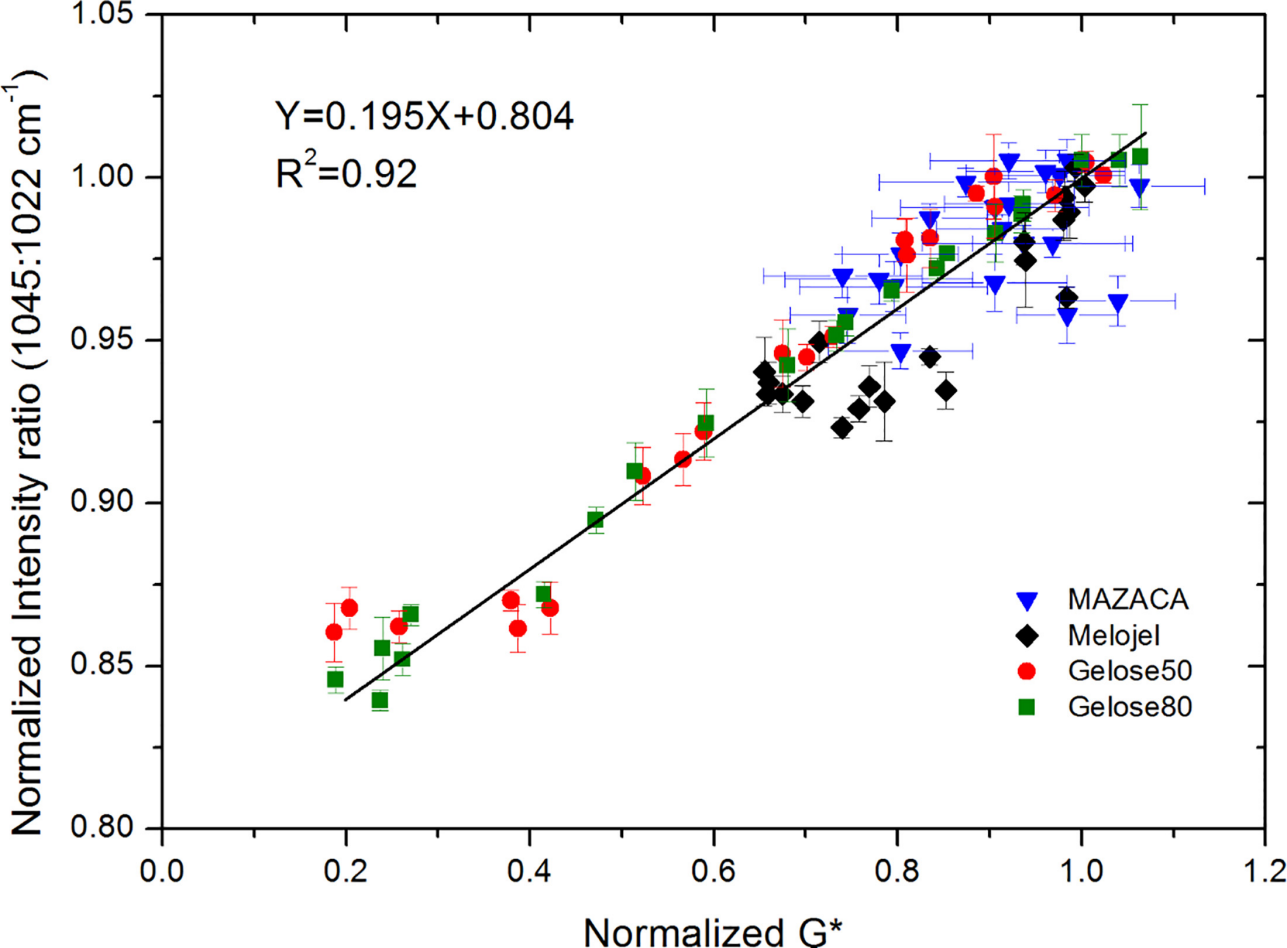
Relationship between the normalized G* (relative to the plateau value) and the normalized FTIR intensity ratio (1045: 1022 cm^-1^) for all maize starches during retrogradation.

For the high amylose maize starches, Gelose 50 and Gelose 80, the G* and the 1045/1022 cm^-1^ ratio reached a plateau after 15–20 days. However, for normal maize starch (Melojel), the G* and the 1045/1022 cm^-1^ ratio reached a plateau around 30 days. It is well known that starch retrogradation is composed of two kinetically distinct processes. The rapid retrogradation process involves amylose via the formation of double helical chain segments followed by helix-helix aggregation. This is accompanied by a slow recrystallization of the short amylopectin chains [43, 44]. The initial rapid rise of both G* and the 1045/1022 cm^-1^ ratio in high amylose maize starch Gelose 50 and Gelose 80 is probably due to the short-range ordering of amylose [43]. In contrast, the slow amylopectin crystallization is believed to contribute mainly to the long term retrogradation [28, 45]. The retrogradation in high amylose starch (Gelose 50 and Gelose 80) and normal maize starch (Melojel) however, is due to the synergistic interactions between amylose and amylopectin [46]. It is suggested that at low amylopectin content, the amylose component functions as a nuclei and/or it co-crystallizes with the amylopectin in some degree [46]. It is also possible that the aggregation of amylose could result in less water available for amylopectin molecules, thus causing the amylopectin clusters to combine. [2]. The G* and value of 1045/1022 cm^-1^ ratio of waxy maize starch (MAZACA) does not increase remarkably with storage time, signalling the lowest degree of retrogradation. It is suggested that when low concentration waxy maize starch (i.e., less than 30%) is gelatinized, the amylopectin clusters are relatively far apart, making it difficult for them to re-associate [2]. This result is in agreement with other studies on the retrogradation of waxy maize starch after heat or high pressure treatments [14] and when submitted to extrusion treatment [39]. By plotting the normalized G* vs the normalized 1045/1022 cm^-1^ ratio to their plateau values (relative change) for all maize starch with all pressure releasing rate, a universal linear relationship between these two parameters is found (Fig 5). The results demonstrate that there is a general linear relationship between the development of retrograded gel strength and the evolution of its short-range ordered structures. This linear relationship can be explained by assuming that the normalized 1045/1022 cm^-1^ ratio is an indication of the extent of retrogradation and is related to the volume fraction *ϕ* occupied by the retrograded starch molecules. These retrograded molecules are expected to have a complex modulus G*_ret_ higher than G*_0_ of the non-retrograded starches, since they form crystalline structures. The complex modulus of the starch pastes can be approximated by either equations (Considine, et al. 2011):

$$G^{*}=(1-\phi )G_{0}^{*}+\phi G_{ret}^{*}$$ (1)

$$\frac{1}{G^{*}}=\frac{\left(1-\phi \right)}{G_{0}^{*}}+\phi /G_{ret}^{*}$$ (2)

Both Eqs (1) and (2) will yield a linear relationship between G* and *ϕ* (i.e. the normalized 1045/1022 cm^-1^ ratio) for small values of *ϕ*, and for small variations in *ϕ* as indicated by the FTIR measurements.

## Conclusions

Rheological methods and FTIR measurements can be employed to characterize the retrogradation behaviour of maize starch with various amylose content during storage at 4°C after high pressure treatment. FTIR results demonstrate that the amount of short-range crystallization in starch increases, while the dynamic rheological experiments reveal an increase of G* as a function of storage time. The rheological and FTIR analysis of HHP treated starches demonstrate that retrogradation does not depend on the pressure releasing rate. Furthermore, the speed of retrogradation of high amylose starch is higher than that of normal maize starch. This is probably due to the fact that the amylose crystallization accounts for the starch short-term retrogradation, while amylopectin crystallization mainly contribute to the starch long-term retrogradation. Note that this study was carried out at a starch concentration of around 10%: w/w and we expect that the starch retrogradation during storage will depend on this concentration and water content. Overall, the results of FTIR and rheological studies provide complementary information on the retrogradation behaviour of HHP treated maize starch with various amylose content under conditions relevant to food storage conditions. The finding of this study can be used to improve the physical stability and textural characteristics of HHP processed starch-rich food products. We wonder if this behavior such as the distribution of phases is related to the complex elastic modulus of other materials such as other polymer blends; biological and non-biological such as peptides aggregations. Finally while a HHP of 600 MPa is applied in this study, and a maximum release time of 12 min (slowest release rate was 50 MPa/min), it will be worth investigating if ultra-HHP (>GPa) and slower HHP release rates will confirm if the rate of starch degradation remain independent of the HHP release rate.
